# Adherence to a “MediterrAsian” diet is associated with weight loss-independent improvements in liver fat and lipid profile, but not glucoregulation or inflammation: secondary analysis of a randomized controlled trial

**DOI:** 10.3389/fnut.2025.1623612

**Published:** 2025-07-24

**Authors:** Yu Chung Chooi, Faidon Magkos, Jadegoud Yaligar, Navin Michael, Suresh Anand Sadananthan, Yeshe Manuel Kway, S. Sendhil Velan, Kevin Junliang Lim, Xianning Lai, Long Hui Wong, Yap Seng Chong, Evelyn Xiu Ling Loo, Johan G. Eriksson

**Affiliations:** ^1^Institute for Human Development and Potential (IHDP), Agency for Science, Technology and Research (A*STAR), Singapore, Singapore; ^2^Department of Nutrition, Exercise, and Sports, University of Copenhagen, Frederiksberg, Denmark; ^3^Department of Medicine, Yong Loo Lin School of Medicine, National University of Singapore (NUS), Singapore, Singapore; ^4^WIL@NUS Corporate Laboratory, Center for Translational Medicine, National University of Singapore (NUS), Singapore, Singapore; ^5^Department of Obstetrics and Gynaecology, Yong Loo Lin School of Medicine, National University of Singapore (NUS), Singapore, Singapore; ^6^Human Potential Translational Research Programme, Department of Paediatrics, Yong Loo Lin School of Medicine, National University of Singapore (NUS), Singapore, Singapore; ^7^Department of General Practice and Primary Health Care, University of Helsinki and Helsinki University Hospital, Helsinki, Finland; ^8^Folkhälsan Research Center, Helsinki, Finland

**Keywords:** diet quality, diet treatment, intrahepatic triglyceride, non-alcoholic fatty liver disease, metabolic dysfunction-associated steatotic liver disease

## Abstract

**Background:**

Greater adherence to the Mediterranean Diet (MedDiet) has been associated with improved inflammatory biomarkers in Western populations, suggesting that the anti-inflammatory effect is crucial for improvements in body weight, body composition, and cardiometabolic risk factors observed with the MedDiet. We previously reported that a calorie-restricted MedDiet adapted to the Asian food culture has beneficial effects on body composition, liver fat, and cardiometabolic risk markers in Chinese women with fatty liver disease.

**Objective:**

To evaluate the effects of MedDiet on inflammation and examine the relationship between dietary adherence and changes in health outcomes.

**Methods:**

88 non-diabetic Chinese women with fatty liver who participated in a 3-arm, 12-week dietary randomized controlled trial were included in this secondary analysis. Adherence to the MedDiet was assessed using a validated questionnaire. Correlation analysis was performed to identify the relationships between changes in total and food group-specific MedDiet scores and changes in anthropometric measures, body fat percentage, liver fat, muscle and abdominal fat, as well as cardiometabolic and inflammation markers from baseline to post-intervention. Analyses were conducted both without adjustments and after adjusting for weight change.

**Results:**

Body weight, total body fat, visceral and subcutaneous adipose tissues, and liver fat decreased significantly after the intervention, in tandem with improvements in markers of glucose and lipid metabolism and inflammation. The change in MedDiet scores (total and individual food groups) correlated with changes in liver fat and improvements in lipid profile, but not with changes in measures of glucose regulation and inflammation after adjusting for changes in body weight. Increased intake of vegetables, nuts, fish, legumes and olive oil appears to be the main driver of these associations.

**Conclusion:**

Greater adherence to the MedDiet among nondiabetic Asian women with fatty liver is associated with greater improvements in hepatic fat and lipid profile, but the association with glucose regulation and inflammation is less pronounced. It thus remains unclear whether resolution of inflammation is the key mechanism for the health benefits of MedDiet.

**Clinical trial registration:**

clinicaltrials.gov, identifier [NCT05259475].

## Introduction

Metabolic dysfunction–associated steatotic liver disease (MASLD), previously known as non-alcoholic fatty liver disease (NAFLD), is the most common chronic liver disease affecting approximately 25% of all adults worldwide (1, 2). Lifestyle factors and particularly dietary habits are thought to play an important role in the etiology of MASLD. Excess consumption of calories but also diets rich in fructose and sugar-sweetened beverages, high in saturated fat, and low in unsaturated omega-3 and omega-6 fatty acids have been associated with the development and progression of MASLD (3, 4).

Weight loss through calorie restriction decreases hepatic fat content in a dose-dependent manner, and improves liver histology (5, 6). Much less information is available about the role of diet quality in improving MASLD (7). The Mediterranean Diet (MedDiet) is widely recognized as a healthy and sustainable eating pattern, based on staple foods that are produced locally in the Mediterranean region. This diet is characterized by a high intake of plant-based foods (vegetables, legumes, fruits, nuts, and cereals such as wholegrain), along with a moderate consumption of wine, fish and dairy, and a high intake of monounsaturated fatty acids (olive oil as the main source of fat), in lieu of saturated and trans fatty acids and processed carbohydrates (8, 9). Many longitudinal and interventional studies have demonstrated that the MedDiet improves metabolic risk factors and the incidence of cardiometabolic diseases, including MASLD, typically in proportion to dietary adherence (10–16).

Studies have also consistently shown an inverse association between adherence to the MedDiet and a wide array of inflammatory biomarkers (17–19). Inflammation is essential for protecting the body against infection or injury, but pathological inflammation is linked to tissue damage and disease (20). Chronic subclinical low-grade inflammation is believed to play a significant role in the pathophysiology of obesity-related comorbidities, including type 2 diabetes and MASLD (21). Accordingly, the health-promoting effects of the MedDiet are believed to be at least partly due to its antioxidant and anti-inflammatory properties (9, 22–24). This data, however, comes mostly from Western populations (25, 26). Many of the traditional MedDiet foods are not relevant or not easy to incorporate in the Asian food culture.

We have previously adapted the MedDiet for Asians, using locally available and acceptable foods, and reported that such a “MediterrAsian” diet improves body composition, liver fat, and cardiometabolic markers in Chinese women with MASLD (27). In this secondary analysis, we computed the degree of adherence to the MedDiet reflected by dietary scores that consider both the frequency and amount of consumption of relevant foods (28) and examined its relationship to the observed changes in body composition, liver fat, cardiometabolic risk factors and inflammatory markers.

## Methods

### Study design

The TANGO (ecTopic fAt in siNGaporean wOmen - the culprit leading to gestational diabetes, metabolic syndrome, and type 2 diabetes) study was a 12-week double-blinded, parallel-design randomized controlled trial (RCT) that examined the health effects of a calorie-restricted, Asian-adapted MedDiet, with (*ni =* 31) or without (*ni =* 28) pentadecanoic acid supplementation, against a standard hypocaloric control diet (*ni =* 29). For the purposes of this secondary analysis, groups were collapsed, and individuals were ranked based on their MedDiet adherence change score during the intervention.

The details of the study design, randomization, and results on cardiometabolic risk factors have been published previously (27). Briefly, Chinese women aged 21–45 years who had a body mass index (BMI) between 23 and 35 kg/m^2^ and MASLD were recruited from the community between October 2021 and March 2022 (Supplementary Figure 1). At the time this study was designed and carried out, the term MASLD had not been introduced yet; NAFLD was thus assessed by ultrasound imaging, as a controlled attenuation parameter ≥268 dB/m (FibroScan 502, Echosens, Paris, France). Enrolled participants had no prior history of diabetes mellitus, other than gestational diabetes, and all had non-diabetic fasting plasma glucose (<7.0 mmol/L) and glycated hemoglobin (HbA_1C_ < 6.5%) concentrations during screening. Participants with significant organ system dysfunction or disease (e.g., hepatic, renal, or thyroid dysfunction), those who were pregnant, lactating, consuming alcohol regularly (≥4 days/week, or ≥6 drinks/week), those using medications known to affect metabolism or gut microbiota (e.g., antibiotics and oral contraceptives), and those suffering from severe diarrhea and recent weight loss (≥5% over the past 3 months) were excluded. Ethics approval was obtained from the Domain Specific Review Board of the National Healthcare Group in Singapore, and the study was conducted in accordance with relevant guidelines. All participants provided signed informed consent prior to enrolment.

### Diet intervention

All participants received counselling from a research dietitian focusing on making healthier food choices and reducing total energy intake to facilitate weight loss. They were recommended to consume moderately low-calorie diets (1,000–1,500 kcal/day) during the 12-week intervention, estimated to induce an energy deficit of 500–1,000 kcal/day relative to energy needs for weight maintenance. Daily energy requirements were calculated by multiplying the measured resting metabolic rate by a physical activity level of 1.3, as all participants were sedentary. Standard measurement cups were provided to facilitate better management of portion size. Dietary counselling also aimed at promoting healthy eating habits based on the My Healthy Plate from the Singapore Health Promotion Board (29), focusing on consuming adequate amounts of fruits and vegetables, fish (≥2 portions weekly), choosing wholegrain products instead of refined ones, choosing low-fat options for dairy products (milk, yoghurt, cheese) and lean meat products, using healthier oils (e.g., olive oil) instead of butter and oils rich in saturated fat, limiting added sugar intake, and minimizing intake of ruminant meat (beef, lamb) and butter.

Participants assigned to the MedDiet groups received, in addition to the general dietetic advice, nutrition education on MedDiet and food components and were required to consume 12 frozen study meals per week, and soymilk once daily (with or without 300 mg of pentadecanoic acid) throughout the 12-week intervention. The 12 frozen meals (providing an average of 350 kcal each, with 36% of energy from carbohydrate, 21% from protein, 33% from monounsaturated (MUFA) and monounsaturated (PUFA) fatty acids; and 7 g of fiber) were prepared in line with the Asian cuisine. The calorie content of the soymilk supplement was 108 kcal (38% of energy from carbohydrate, 22% from protein, 31% from MUFA and PUFA; and 4 g of fiber). The frozen meals and soymilk were sourced and produced in a single batch and provided by Wilmar International Ltd. (Singapore). Almonds, frozen vegetables, frozen soy-based protein, oat bran, millet and olive oil were provided to the two MedDiet groups. All these food items, except the frozen meals as such, are available in grocery stores in Singapore although they are not particularly popular in the local food culture. The overall Asian-adapted MedDiet was high in fiber, MUFA and PUFA, wholegrain products, legumes, vegetables, salmon, plant-based protein, nuts, fruits and high-polyphenol extra virgin olive oil.

To encourage compliance, participants were contacted by phone after the first 2 weeks and met with a research dietitian every 4 weeks. Adherence to the dietary intervention was evaluated by meal checklists completed daily by the participants in the two diet groups. No frozen meals or soymilk were provided to control participants, but they had access to the dietitian consultations focusing on healthier food choices and weight loss during their monthly visits. Almost 80% of the participants in the control group opted to meet with dietitians during their monthly visits.

### Mediterranean diet adherence

A validated 14-item MedDiet questionnaire was used to assess the degree of adherence to the Asian-adapted MedDiet (28), but we eventually included only 11 of the 14 items in the analysis as three items were deemed irrelevant for the Asian framework (frequency of using sofrito—a sauce made with tomato and onion, garlic, leek and simmered with olive oil; wine consumption; and preference towards chicken, turkey or rabbit meat instead of veal, pork, hamburger or sausage). Each item is scored from 0 to 1, yielding a maximum score of 11. Adherence was computed by the research dietitian using 3-day food diaries (two weekdays and one weekend), simple dietary questionnaires probing for the frequency of consumption of various food groups (vegetables, fruits, wholegrain, dairy, sweetened drinks, desserts, unhealthy snacks and high fat foods), or weekly meal checklists. The change scores (post-intervention minus baseline) were then calculated and used for analysis.

### Clinical visits and outcome assessment

The participants completed four clinical visits for study-related measurements at weeks 0 (baseline), 4, 8 and 12 (end of intervention); for the purposes of this analysis, only the baseline and end-of-study visits were used. At each visit, participants arrived at the Human Development Research Centre at the National University of Singapore (NUS) campus in the morning, after having fasted overnight. Body weight was measured, and fasting blood samples were collected on all four visits. Resting Metabolic Rate, liver fat content, body composition and body fat distribution were measured at baseline and at the end of the intervention.

Anthropometric parameters (weight, height, hip and waist circumferences) and systolic and diastolic blood pressures were measured according to routine standardized procedures. Fat mass and fat-free mass were determined by bioelectrical impedance analysis (Impedimed, SFB7, Brisbane, Australia). Blood was collected through venipuncture after 10–12 h of fasting. Fasting glucose, insulin, HbA_1C_, total plasma triglyceride, total cholesterol, low-density lipoprotein (LDL) and high-density lipoprotein (HDL) cholesterol concentrations were determined by standard methods at the National University Hospital (NUH) Referral Laboratory (accredited by the College of American Pathologists). Cytokine concentrations were determined by the OLINK target 48 assay at the Viral Research and Experimental Medicine Centre at SingHealth Duke-NUS (ISO accreditation).

Intra-abdominal fat (visceral adipose tissue, VAT) and subcutaneous abdominal adipose tissue (SAT) volumes were determined by magnetic resonance imaging (MRI) using two-point Dixon fat-water imaging and body matrix coil on Siemens Prisma 3 T MR scanner (Siemens Healthcare, Erlangen, Germany). A deep learning based automatic segmentation algorithm followed by manual editing was used to delineate and quantify the VAT and SAT compartments (30). Liver fat content was determined using multi echo Dixon fat-water imaging sequence. Multiple regions of interest (ROIs) were selected within the liver, carefully excluding blood vessels and boundaries, and liver fat was quantified as the mean proton density fat fraction (PDFF) within the selected ROIs (31). Skeletal muscle fat content in the soleus muscle was determined using magnetic resonance spectroscopy. The spectrum was quantified using LCModel (32) and the amount of intramyocellular lipids (IMCL) was calculated and expressed as a ratio with respect to water and corrected for transverse relaxation time (T2) losses (33).

### Statistical analysis

Data analysis was conducted based on the intention-to-treat principle with last-observation-carried-forward (LOCF) for imputing missing data for 5 participants (4 discontinued intervention during the study and 1 declined blood sampling after the baseline visit). Data was analyzed with SPSS version 26 (IBM SPSS, Chicago, IL). The Shapiro–Wilk test was used to evaluate the distribution of data. Differences between baseline and end-of-study were assessed using the paired Student’s t test for normally distributed variables or the Wilcoxon signed rank test for non-normally distributed variables. Associations between variables of interest were evaluated using correlation analysis (Pearson or Spearman), visualized as heat maps. Analyses were conducted both without adjustments and after adjusting for weight change. Results are reported as means ± SDs or medians with quartiles (quartile 1, quartile 3). Statistical significance was set at *P* < 0.05.

## Results

### Participants

In total, 255 Chinese women living in Singapore were assessed for eligibility and 90 of them were enrolled and randomized. Of them, 88 participants (diet with pentadecanoic acid, *ni =* 31; diet without pentadecanoic acid, *ni =* 28; control, *ni =* 29) attended the baseline visit (week 0) and 84 completed the study (Supplementary Figure 1). At baseline, participants had a mean age of 35.7 years and a mean BMI of 28.4 kg/m^2^. Other characteristics at baseline are shown in Table 1.

**Table 1:** Body composition, fat distribution, and cardiometabolic and inflammatory markers before and after the diet interventions.

	Baseline	Week 12	*p*-value
Body composition			
Weight (kg)	74.0 ± 8.9	71.0 ± 8.8	<0.001
Fat mass (kg)	26.3 ± 5.3	24.0 ± 5.2	<0.001
Waist circumference (cm)	90.5 ± 7.4	88.0 ± 6.8	<0.001
VAT (cc)	1,802 ± 572	1,623 ± 549	<0.001
SAT (cc)	3,824 (3,400; 4,931)	3,529 (3,021; 4,401)	<0.001
IMCL/Water (%)	1.3 (0.9; 1.7)	1.4 (1.0; 1.8)	0.743
Liver PDFF (%)	10.2 (5.6; 17.7)	7.1 (3.3; 10.9)	<0.001
Cardiometabolic			
Systolic BP (mmHg)	122 ± 12	117 ± 11	<0.001
Diastolic BP (mmHg)	79 ± 8	76 ± 8	<0.001
HbA_1C_ (%)	5.4 (5.2; 5.7)	5.3 (5.1; 5.5)	<0.001
Fasting plasma glucose (mmol/L)	5.0 ± 0.5	4.8 ± 0.4	<0.001
Insulin (mU/L)	11.0(8.0; 13.7)	8.0 (5.9; 11.0)	<0.001
HOMA-IR	2.4 (1.8; 3.2)	1.7 (1.2; 2.3)	<0.001
Total cholesterol (mmol/L)	5.3 (4.8; 6.0)	5.1 (4.3; 5.5)	<0.001
HDL-cholesterol (mmol/L)	1.4 (1.2; 1.6)	1.3 (1.2; 1.5)	<0.001
LDL-cholesterol (mmol/L)	3.4 ± 0.8	3.1 ± 0.8	<0.001
Triglycerides (mmol/L)	1.1 (0.8; 1.4)	1.0 (0.7; 1.2)	0.008
Inflammation			
CRP (mg/L)	2.2 (1.1; 4.8)	1.8 (0.8; 4.5)	0.031
OLR1 (pg/mL)	230 (134; 352)	153 (95; 276)	<0.001
IL-1β (pg/mL)	0.050 (0.024; 0.092)	0.039 (0.020; 0.081)	0.017
IL-2 (pg/mL)	0.011 (0.010; 0.012)	0.012 (0.011; 0.014)	0.002
IL-4 (pg/mL)	0.041 (0.038; 0.045)	0.043 (0.040; 0.046)	0.008
IL-6 (pg/mL)	3.0 (2.0; 4.9)	2.4 (1.8; 3.7)	0.001
IL-10 (pg/mL)	4.6 (3.8; 6.5)	4.4 (3.1; 6.0)	0.009
HGF (pg/mL)	571 (449; 685)	443 (369; 588)	<0.001
TNF-α (pg/mL)	16.8 (14.3; 19.9)	15.7 (13.4; 18.8)	0.013
VEGFA (pg/mL)	748 (528; 1,043)	614 (429; 912)	<0.001
EGF (pg/mL)	268 (196; 398)	218 (130; 326)	0.001
OSM (pg/mL)	5.7 (3.8; 7.5)	4.5 (3.4; 6.9)	<0.001

Values are means ± SDs or medians (quartile 1; quartile 3) for 88 participants (per ITT). BP, blood pressure; CRP, C-reactive protein; EGF, epidermal growth factor; HbA_1C_, glycated hemoglobin; HDL, high density lipoprotein; HGF, hepatocyte growth factor; IMCL, intramyocellular lipid; IL-1β, interleukin-1 beta; IL-2, interleukin-2; IL-4, interleukin-4; IL-6, interleukin-6; IL-10, interleukin-10; LDL, low density lipoprotein; OLR1, oxidized low-density lipoprotein receptor 1; OSM, oncostatin M; PDFF, proton density fat fraction; SAT, subcutaneous abdominal adipose tissue; TNF-α, tumor necrosis factor alpha; VAT, visceral adipose tissue; VEGFA, vascular endothelial growth factor A.

### Changes in MedDiet scores and inflammatory markers

In response to the two MedDiet interventions, there was a significant increase in the total MedDiet score: from 4.47 to 8.27 after the MedDiet with pentadecanoic acid and from 4.59 to 8.27 after the MedDiet without pentadecanoic acid (both *p ≤* 0.001). The total MedDiet score did not change in the control group (from 4.63 to 4.72, *p* = 0.383). As a whole, participants increased their intake of vegetables, fruits, nuts, fish, legumes, and olive oil; and decreased their consumption of sugar-sweetened beverages, desserts and pastries, and red meat (Table 2). The only individual MedDiet food score that did not change was that of butter consumption.

**Table 2:** Adherence to the Mediterranean diet before and after the diet interventions.

	Baseline	Week 12	*p*-value
Total MedDiet score	4.56 ± 0.92	7.10 ± 2.14	<0.001
Consumption ≥ 2 servings/day of vegetables	0.50 (0.33; 0.73)	1.0 (0.5; 1.0)	<0.001
Consumption ≥ 3 fruit units/day (including natural fruit juice)	0.15 (0.15; 0.25)	0.25 (0.15; 0.33)	<0.001
Consumption ≥ 3 servings/week of nuts (1 serving 30 g)	0.0 (0.0; 0.0)	0.06 (0.0; 1.0)	0.002
Consumption ≥ 3 servings/week of fish or shellfish	0.33 (0.33; 0.66)	0.66 (0.56; 0.66)	<0.001
Consumption ≥ 3 servings/week of legumes	0.10 (0; 0.18)	0.56 (0.15; 0.66)	<0.001
Consumption < 1 serving/day of sweetened or carbonated beverages	0.75 (0.5; 1.0)	0.83 (0.75; 1.0)	<0.001
Consumption < 3 times/week of commercial sweets or pastries	0.75 (0.5; 1.0)	1.0 (0.67; 1.0)	<0.001
Consumption < 1 serving/day of red meat, hamburger or processed meat products	0.50 (0.5; 0.75)	0.75 (0.5; 0.75)	<0.001
Using olive oil as main culinary fat	0.0 (0; 1.0)	1.0 (0.52; 1.0)	<0.001
Consumption ≥ 4 tablespoons/day of olive oil	0.0 (0; 0)	0.83 (0; 1.0)	<0.001
Consumption < 1 serving/day of butter, margarine or cream (1 serving 12 g)	1.0 (1.0; 1.0)	1.0 (1.0; 1.0)	0.655

Values are means ± SDs or medians (quartile 1; quartile 3) for 88 participants (per ITT). MedDiet, Mediterranean diet.

In response to the diet interventions, circulating markers of inflammation generally decreased (Table 1 and Supplementary Table 1), with little evidence of a differential response among groups (time-by-group interactions), albeit the improvements in some markers were or tended to be of lesser magnitude in the control group than in the two MedDiet groups (Supplementary Table 2). Out of the OLINK panel, we selected the most relevant and frequently reported markers reported in studies of diet–induced changes in inflammation (34) for our downstream analysis.

### Changes in health outcomes in relation to MedDiet score

For the group as a whole, body weight, body fat mass, waist circumstance, liver fat, VAT and SAT improved significantly after the dietary intervention, in conjunction with improvements in multiple cardiometabolic risk factors and widespread reductions in markers of inflammation (Table 1). Overall, the participants lost 4.0 ± 3.7% of their baseline body weight and liver PDFF decreased by 24.5 ± 28.1% (relative changes from baseline).

**Figure 1:**
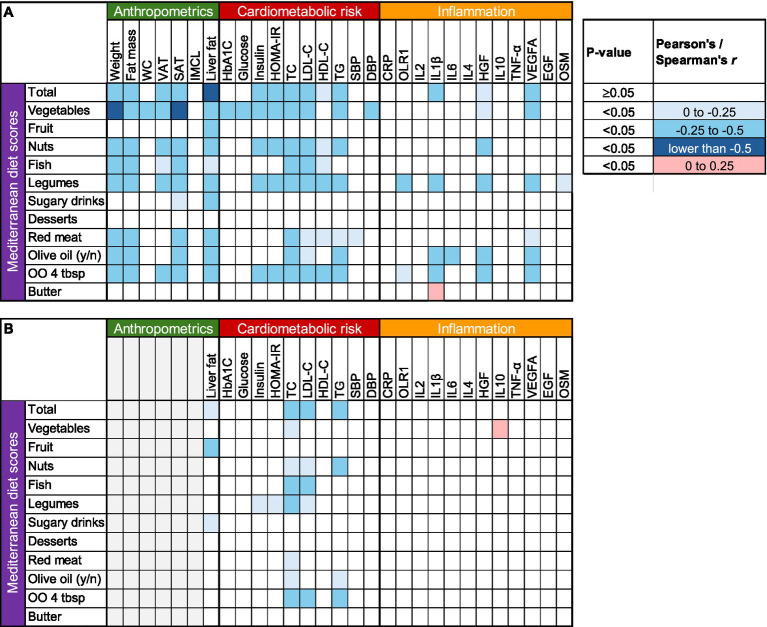
Correlation heat map between changes in Mediterranean Diet scores and changes in anthropometric, cardiometabolic and inflammatory parameters. **(A)** Unadjusted, **(B)** adjusted for weight change. CRP, C-reactive protein; DBP, diastolic blood pressure; EGF, epidermal growth factor; HbA_1C_, glycated hemoglobin; HDL, high density lipoprotein; HGF, hepatocyte growth factor; IMCL, intramyocellular lipid; IL1 *β*, interleukin-1 beta; IL2, interleukin-2; IL4, interleukin-4; IL6, interleukin-6; IL10, interleukin-10; LDL, low density lipoprotein; OLR1, oxidized low-density lipoprotein receptor 1; OO, Olive oil; OSM, oncostatin M; PDFF, proton density fat fraction; SAT, subcutaneous abdominal adipose tissue; SBP, systolic blood pressure; TC, total cholesterol; TG, triglycerides; TNF-*α*, tumor necrosis factor alpha; VAT, visceral adipose tissue; VEGFA, vascular endothelial growth factor A; WC, waist circumference.

The heat map in Figure 1A depicts the unadjusted associations between changes in MedDiet score and changes in anthropometric, metabolic, and inflammatory markers. Greater intake of vegetables, nuts, fish, legumes, and olive oil, coupled with reduced red meat consumption, appeared to drive most of these associations. Greater adherence to the MedDiet was strongly associated with reductions in weight and various body fat compartments including liver fat; significantly associated with decreases in total and LDL-cholesterol and triglyceride concentrations; but not associated with improvements in inflammatory markers except for hepatocyte growth factor (HGF), interleukin-1 beta (IL1 *β*) and vascular endothelial growth factor A (VEGFA).

Because weight loss itself is directly associated with changes in body fat compartments, liver fat, and cardiometabolic risk factors (35), we also examined these relationships after adjusting for body weight changes. Increased adherence to the MedDiet was still associated with reduced liver fat and improved lipid profile, but not with changes in glucoregulation, blood pressure, or inflammation after this adjustment (Figure 1B).

## Discussion

In this study, greater adherence to an Asian-adapted MedDiet was significantly associated with greater weight loss and greater reductions in fat mass, liver fat, VAT, SAT, and plasma cholesterol (total and LDL) and triglyceride concentrations. However, after adjusting for changes in body weight, improvements in glucose metabolism and markers of inflammation were no longer associated with MedDiet adherence (Figure 1). These data suggest that the anti-inflammatory effects of the MedDiet (36) are most likely driven by the accompanying reductions in body weight and body fat (37, 38).

Inflammation is a normal defense mechanism for protecting the body against infection and injury, but when it persists, the mediators released by activated immune cells can cause tissue damage and contribute to the development of various diseases (20). Chronic inflammation is characterized by elevated levels of circulating inflammatory markers (20), and many studies have reported that the MedDiet can resolve inflammation and oxidative stress (17–19, 39, 40). These observations have been taken as suggestive that the antioxidant and anti-inflammatory properties of the MedDiet are critically important for the observed health benefits (9, 22–24, 26). In our study, however, the improvements observed in most biomarkers of inflammation were generally not associated with MedDiet adherence. The duration of our study was relatively short, and possibly inadequate for more pronounced changes in inflammatory markers to manifest (34). Nevertheless, certain markers of inflammation, such as C-reactive protein, decrease in response to dietary interventions within 3 months, whereas others require more time to change (34). In unadjusted analysis (Figure 1A), the inverse relationship with both HGF and VEGFA (i.e., greater diet adherence correlated with greater reductions in these molecules) likely reflects a coordinated downregulation of angiogenesis (41, 42), rather than an anti-inflammatory effect, as there were no relationships with most proinflammatory cytokines and pro-oxidants that are known to increase VEGFA expression, such as interleukins 1β and 6, oncostatin M, and tumor necrosis factor-*α* (43), whereas HGF itself is an anti-inflammatory cytokine (44). These results do not support the notion that resolution of inflammation is a uniform mechanism for the health-promoting benefits of the MedDiet (45). Corroborating this hypothesis, the associations between MedDiet scores and inflammatory molecules largely disappeared in weight change-adjusted analyses (Figure 1B). It is thus likely that weight loss itself, rather than diet composition, mediates the resolution of inflammation (46) and the improvements in some other cardiometabolic risk factors (47). Given this is an association analysis, however, our findings should not be interpreted as evidence of causal relationships.

Among the individual food groups, increased consumption of vegetables, nuts, fish, olive oil, and legumes, and decreased consumption of red meat showed the strongest correlations with improvements in body weight and body composition, liver fat, and cardiometabolic risk factors (Figure 1). The associations with liver fat and lipid profile were only little attenuated after adjusting for weight change, suggesting that qualitative characteristics of the MedDiet mediate these benefits, and not merely weight loss. Interestingly, increased fruit consumption and decreased intakes of foods rich in simple sugars (sugar-sweetened beverages, desserts and pastries) were not significantly associated with the health benefits of MedDiet. Vegetables are particularly rich in vitamins (especially C and A), minerals (especially electrolytes), and phytochemicals (especially antioxidants and anti-inflammatory compounds such as carotenoids, flavonoids and polyphenols) (48). However, we did not observe any particularly strong associations between the increase in vegetable consumption and improvements in circulating inflammatory markers. Accordingly, it is likely that the increased dietary fiber content of vegetables is more important for the observed health benefits in the context of our study. Dietary fiber alters transit time in the small intestine and delays gastric emptying, thereby increasing satiety, and may also result in more energy being lost in the feces (49–51). Soluble fiber in vegetables also lowers circulating cholesterol by increasing fecal excretion of bile salts (49), consistent with the greater reductions in circulating total and LDL cholesterol in participants who experienced greater increases in vegetable intake.

The traditional MedDiet is characterized by high intakes of olive oil (rich in MUFA), nuts, fruits, vegetables and fish and low intakes of red meat, dairy products and added sugars; and wine in moderation together with meals (8). Our experimental diets were designed to have similar characteristics, i.e., rich in MUFA, PUFA, and fiber, and were adapted culturally to the local Asian cuisine. Thereby, several characteristics of the Mediterranean diet can be incorporated in an Asian context based on regional food availability and aligned with local food environment and traditional eating habits. Such a local adaptation is important not only for facilitating dietary adherence and scalability, but also for ensuring sustainability and national food security. Not surprisingly, the provision of experimental foods (walnuts, almonds, and hazelnuts or olive oil) free-of-charge to study participants increases intake of these foods, more so than a simple diet prescription or advice (52). This approach aligns with behavioral economics and “nudging” principles, such as utilizing the path of least resistance, establishing desirable defaults, and increasing the availability of healthy food options (53). We successfully applied these strategies in our study by providing our participants two frozen meals daily with almonds, frozen vegetables, frozen soy-based protein, oat bran, millet and olive oil—i.e., shifting the quality of the diet to a more Mediterranean-like pattern. Some foods such as olive oil and almonds (and nuts, in general) are not common in the Asian food culture, and increasing their intake presents many challenges. Providing free access to study foods combined with nutrition education significantly increased intake for the duration of the study, but we cannot attest to the long-term sustainability of this dietary paradigm. More studies are needed to better understand the factors involved in long-term adherence or to find alternative food groups that are better aligned with the Asian food culture (54). Future research should explore the feasibility and consumer acceptability of a “MediterrAsian” diet, taking into account regional dietary preferences, cultural norms, food availability, and culinary practices. There are potentially several largely unexplored components of traditional heritage diets that could be incorporated into local healthy eating patterns (26).

Our study has several strengths but also limitations. While most studies on MedDiet adherence are primarily cross-sectional in design, our study was conducted as an RCT and we examined the associations between changes in adherence and changes in health outcomes after the diet interventions. However, our results cannot inform of cause and effect, neither can they isolate the specific contribution of the Mediterranean-like dietary pattern from the effect of reduced energy intake and weight loss. Moreover, we had to exclude some MedDiet score items from our assessment of adherence, because the original questionnaire developed for Western populations included some questions which were irrelevant for the Asian socio-cultural environment. This does not allow for comparing results of MedDiet adherence scores between studies. Finally, lack of a linear association between MedDiet adherence and resolution of inflammation does not preclude the latter being important in the observed health effects, e.g., if there is a threshold effect that was achieved in most participants. Our study also shows that cultural factors and ethnicity need to be considered when discussing health effects of various dietary paradigms, as findings from Western populations cannot be directly extrapolated or generalized to Asian populations.

## Conclusion

In conclusion, greater adherence to an Asian-adapted MedDiet is associated with greater reductions in liver fat and cardiovascular risk factors, but not with improvements in glucose regulation and inflammation after adjusting for changes in body weight. Increased consumption of vegetables, nuts, fish, legumes and olive oil appear to mediate most of these associations. Future studies should explore how to substitute key MedDiet foods with palatable foods that are widely accepted in Asia, or how to make these MedDiet foods more readily available throughout the region.

## Data Availability

The original contributions presented in the study are included in the article/Supplementary material, further inquiries can be directed to the corresponding authors.

## Glossary

BP: blood pressure
CRP: c-reactive protein
EGF: epidermal growth factor
HbA_1C_: glycated hemoglobin
HDL: high density lipoprotein
HGF: hepatocyte growth factor
IMCL: intramyocellular lipid
IL-1β: interleukin-1beta
IL-2: interleukin-2
IL-4: interleukin-4
IL-6: interleukin-6
IL-10: interleukin-10
ITT: intention to treat
LDL: low density lipoprotein
LOCF: last observation carried forward
MASLD: Metabolic dysfunction–associated steatotic liver disease
MedDiet: Mediterranean diet
NAFLD: non-alcoholic fatty liver disease
OLR1: oxidized low-density lipoprotein receptor 1
OSM: oncostatin M
PDFF: proton density fat fraction
RMR: resting metabolic rate
SAT: subcutaneous abdominal adipose tissue
TANGO: Ectopic Fat in Singaporean Women - the Culprit Leading to Gestational DiabetesMetabolic Syndrome and Type 2 Diabetes
TNF-*α*: tumor necrosis factor alpha
VAT: visceral adipose tissue;
VEGFA: vascular endothelial growth factor A
