# Supplementary material for: Adherence to a “MediterrAsian” diet is associated with weight loss-independent improvements in liver fat and lipid profile, but not glucoregulation or inflammation: secondary analysis of a randomized controlled trial
Source: Front Nutr. 2025 Jul 24;12:1623612. doi: 10.3389/fnut.2025.1623612 (PMC12330216; doi:10.3389/fnut.2025.1623612)
Supplement: Supplementary file 1 [file Data_Sheet_1.pdf]

Supplementary Material

Adherence to a "MediterrAsian" diet is associated with weight loss-independent improvements in liver fat and lipid profile, but not glucoregulation or inflammation: secondary analysis of a randomized controlled trial

Chooi et al.

Supplemental Figure 1: Flowchart of participants through the study

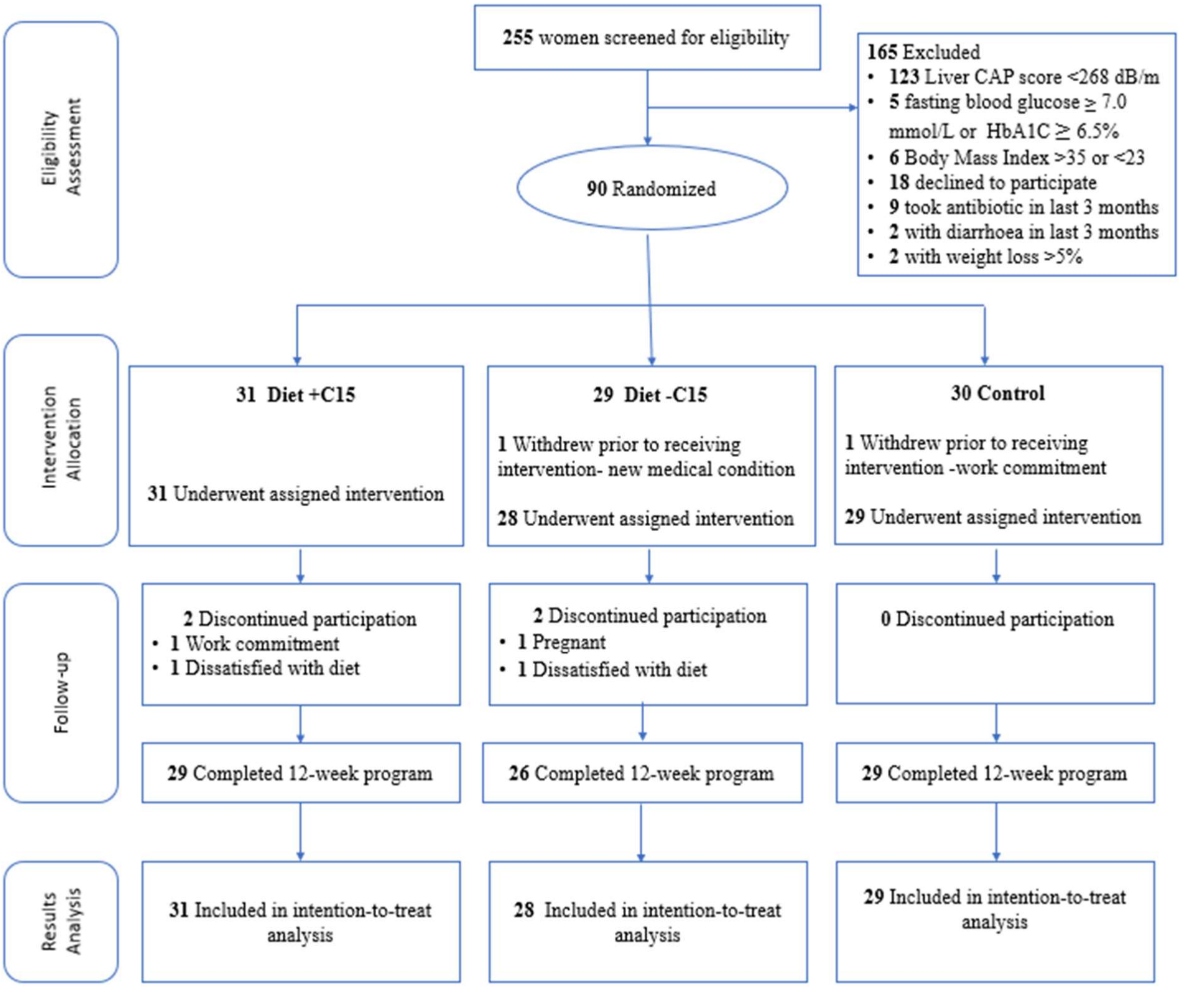

CAP= controlled attenuation parameter, Diet+C15 = Diet with pentadecanoic acid supplementation, Diet-C15= Diet without pentadecanoic supplementation, HbA_{1C} = glycated hemoglobin

Supplementary Table 1: Full OLINK panel of inflammatory markers before and after the diet intervention

	Baseline	Week 12	P-value
**Inflammation**			
CCL2 (pg/mL)	389 (324; 437)	336 (280; 403)	<0.001
CCL3 (pg/mL)	9 (7; 11)	8 (6; 9)	<0.001
CCL4 (pg/mL)	167 (125; 235)	143 (111; 192)	<0.001
CCL7 (pg/mL)	0.88 (0.67; 1.13)	0.89 (0.61; 1.17)	0.241
CCL8 (pg/mL)	71 (54; 95)	61 (45; 80)	<0.001
CCL11 (pg/mL)	88 (69; 106)	77 (64; 95)	0.001
CCL13 (pg/mL)	160 (111; 215)	131 (99; 195)	<0.001
CCL19 (pg/mL)	118 (85; 164)	107 (79; 142)	0.002
CSF1 (pg/mL)	124 (109; 137)	116 (104; 128)	<0.001
CSF2 (pg/mL)	0.14 (0.10; 0.23)	0.15 (0.10; 0.22)	0.448
CSF3 (pg/mL)	120 (84; 156)	123 (99; 143)	0.656
CXCL8 (pg/mL)	10 (8; 13)	10 (8; 12)	0.097
CXCL9 (pg/mL)	34 (26; 45)	35 (26; 43)	0.663
CXCL10 (pg/mL)	89 (68; 126)	81 (63; 111)	0.049
CXCL11 (pg/mL)	65 (49; 81)	60 (45; 80)	0.091
CXCL12 (pg/mL)	193 (170; 227)	195 (165; 222)	0.419
FLT3LG (pg/mL)	100 (86; 129)	93 (77; 117)	<0.001
IFNG (pg/mL)	0.24 (0.17; 0.34)	0.24 (0.15; 0.33)	0.546
IL7 (pg/mL)	7.7 (5.7; 9.2)	7.4 (5.9; 9.3)	0.627
IL13 (pg/mL)	0.36 (0.31; 0.44)	0.38 (0.33; 0.45)	0.552
IL15 (pg/mL)	12.4 (10; 15)	11.7 (10; 15)	0.005
IL17A (pg/mL)	0.40 (0.24; 0.63)	0.41 (0.22; 0.74)	0.643
IL17C (pg/mL)	15 (12; 22)	17 (11; 22)	0.540
IL17F (pg/mL)	0.66 (0.36; 1.52)	0.67 (0.34; 1.39)	0.727
IL18 (pg/mL)	239 (187; 293)	204 (167; 253)	<0.001
IL-27 (pg/mL)	7.2 (4.9; 10.3)	6.4 (4.7; 9.0)	0.322
LTA (pg/mL)	9 (8; 11)	9 (7; 10)	0.068
MPM12 (pg/mL)	203 (172; 259)	208 (164; 279)	0.838
TGFA (pg/mL)	16 (12; 23)	14 (10; 19)	<0.001
TNFSF10 (pg/mL)	506 (423; 601)	490 (407; 565)	0.020
TNFSF12 (pg/mL)	711 (582; 864)	676 (551; 800)	0.006
TSLP (pg/mL)	0.12 (0.06; 0.24)	0.10 (0.04; 0.22)	0.057

Values are medians (quartile 1; quartile 3) for 88 participants (per ITT).

CCL2 = C-C motif chemokine 2, CCL3 = C-C motif chemokine 3, CCL4 = C-C motif chemokine 4, CCL7 = C-C motif chemokine 7, CCL8 = C-C motif chemokine 8, CCL11 = Eotaxin, CCL13 = C-C motif chemokine 13, CCL19 = C-C motif chemokine 19, CSF1 = Macrophage colony-stimulating factor 1, CSF2 = Granulocyte-macrophage colony-stimulating factor, CSF3 = Granulocyte colony-stimulating factor, CXCL8 = Interleukin-8, CXCL9 = C-X-C motif chemokine 9, CXCL10 = C-X-C motif chemokine 10, CXCL11 = C-X-C motif chemokine 11, CXCL12 = Stromal cell-derived factor 1, FLT3LG = Fms-related tyrosine kinase 3 ligand, IFNG = Interferon gamma, IL7 =

Interleukin-7, IL13 = Interleukin-13, IL15 = Interleukin-15, IL17A = Interleukin-17A, IL17C = Interleukin-17C, IL17F = Interleukin-17F, IL18 = Interleukin-18, IL-27 = Interleukin-27, LTA = Lymphotoxin-alpha, MMP12 = Macrophage metalloelastase, TGFA = Protransforming growth factor, TNFSF10 = Tumor necrosis factor ligand superfamily member 10, TNFSF12 = Tumor necrosis factor ligand superfamily member 12, TSLP = Thymic stromal lymphopoietin.

Supplementary Table 2: Selected inflammatory markers before and after the three diet interventions

	Diet + C15 (N=31)		Diet wo C15 (N=28)		Control (N=29)		P-values		
	Baseline	Week 12	Baseline	Week 12	Baseline	Week 12	Time	Diet	Interaction
CRP (mg/L)	2.2 (1.6; 4.9)	2.3 (1.2; 5.1)	2.5 (0.9; 6.2)	2.3 (1.0; 4.6)	1.2 (0.8; 3.6)	1.0 (0.6; 3.1)	0.421	0.196	0.349
OLR1 (pg/mL)	278 (166; 394)	171 (112; 318)	205 (128; 375)	125 (90; 258)	198 (105; 320)	153 (86; 296)	<0.001	0.317	0.173
IL-1β (pg/mL)	0.058 (0.033; 0.107)	0.035 (0.020; 0.095)	0.058 (0.025; 0.085)	0.036 (0.016; 0.059)	0.037 (0.017; 0.081)	0.052 (0.022; 0.102)	0.079	0.460	0.175
IL-2 (pg/mL)	0.011 (0.010; 0.013)	0.012 (0.011; 0.013)	0.011 (0.010; 0.013)	0.012 (0.011; 0.015)	0.011 (0.010; 0.012)	0.012 (0.011; 0.013)	0.061	0.558	0.384
IL-4 (pg/mL)	0.041 (0.038; 0.045)	0.043 (0.040; 0.046)	0.043 (0.040; 0.046)	0.042 (0.039; 0.045)	0.040 (0.036; 0.043)	0.043 (0.041; 0.046)	0.029	0.144	0.019
IL-6 (pg/mL)	3.3 (2.0; 4.9)	3.2 (1.9; 3.8)	4.2 (2.4; 5.9)	2.6 (2.1; 4.3)	2.2(1.8; 3.5)	1.9 (1.6; 2.7)	0.009	0.372	0.990
IL-10 (pg/mL)	4.3 (3.2; 6.6)	3.8 (3.0; 5.6)	5.2 (3.7; 6.9)	4.3 (3.1; 5.8)	4.4 (3.6; 5.4)	4.7 (3.2; 6.1)	0.02	0.382	0.879
HGF (pg/mL)	589 (459; 704)	510 (381; 604)	584 (467; 760)	433 (342; 573)*	476 (401; 654)	443 (371; 594)	<0.001	0.527	0.069
TNF-α (pg/mL)	16.7 (14.5; 19.8)	15.7 (13.9; 19.2)	18.4 (14.4; 21.3)	15.5 (12.9; 18.7)	16.7 (14.2; 19.0)	16.4 (12.8; 19.5)	0.006	0.986	0.253
VEGFA (pg/mL)	731 (527; 902)	593 (428; 761)*	763 (508; 1,084)	607 (403; 942)*	779 (521; 1,179)	759 (430; 1240)	<0.001	0.464	0.009
EGF (pg/mL)	312 (247; 401)	217 (138; 302)	246 (183; 382)	186 (111; 326)	261 (180; 392)	241 (131; 414)	0.001	0.703	0.155
OSM (pg/mL)	5.4 (4.1; 8.9)	4.8 (3.8; 7.2)	5.9 (4.5; 7.5)	4.5 (2.7; 6.3)	5.1 (3.3; 7.2)	4.2 (3.1; 6.8)	<0.001	0.409	0.567

Values are median (quartile 1; quartile 3) for 88 participants (per ITT).
Data were analysed by repeated measures ANOVA (time-by-diet).
*P<0.05 vs baseline in the same group, from Sidak post hoc test.

CRP = C-reactive protein, HGF = hepatocyte growth factor, IMCL = intramyocellular lipid, IL1 β = interleukin-1 beta, IL2 = interleukin-2, IL4 = interleukin-4, IL6 = interleukin-6, IL10 = interleukin-10, OLR1 = oxidized low-density lipoprotein receptor 1, OSM = oncostatin M, TNF-α = tumor necrosis factor alpha, VEGFA = vascular endothelial growth factor A.
